# Assessing heat-related health risk in Europe via the Universal Thermal Climate Index (UTCI)

**DOI:** 10.1007/s00484-018-1518-2

**Published:** 2018-03-15

**Authors:** Claudia Di Napoli, Florian Pappenberger, Hannah L. Cloke

**Affiliations:** 1 Department of Geography and Environmental Science, University of Reading, Reading, UK; 2 Forecast Department, European Centre for Medium-Range Weather Forecasts, Reading, UK; 3 Department of Meteorology, University of Reading, Reading, UK; 4 Department of Earth Sciences, Uppsala University, Uppsala, Sweden; 5 Centre of Natural Hazards and Disaster Science, CNDS, Uppsala, Sweden

**Keywords:** Universal thermal climate index (UTCI), NWP, Thermal health hazard, Heat stress, Mortality, Bioclimatology

## Abstract

In this work, the potential of the Universal Thermal Climate Index (UTCI) as a heat-related health risk indicator in Europe is demonstrated. The UTCI is a bioclimate index that uses a multi-node human heat balance model to represent the heat stress induced by meteorological conditions to the human body. Using 38 years of meteorological reanalysis data, UTCI maps were computed to assess the thermal bioclimate of Europe for the summer season. Patterns of heat stress conditions and non-thermal stress regions are identified across Europe. An increase in heat stress up to 1 °C is observed during recent decades. Correlation with mortality data from 17 European countries revealed that the relationship between the UTCI and death counts depends on the bioclimate of the country, and death counts increase in conditions of moderate and strong stress, i.e., when UTCI is above 26 and 32 °C. The UTCI’s ability to represent mortality patterns is demonstrated for the 2003 European heatwave. These findings confirm the importance of UTCI as a bioclimatic index that is able to both capture the thermal bioclimatic variability of Europe, and relate such variability with the effects it has on human health.

## Introduction

Extreme high temperatures, such as those experienced during a heatwave, represent a serious meteorological hazard to human health and wellbeing. Health impacts include heat-related disorders such as dehydration and sunstroke, and excess morbidity and mortality in affected areas due to cardiovascular and respiratory diseases (Semenza et al. 1996; Curriero et al. 2002). Notable episodes of severe and sustained high temperatures include the 2003 European heatwave and the 2010 Russian heatwave that killed about 70,000 and 55,000 people, respectively (Robine et al. 2008; Barriopedro et al. 2011). With the growing evidence that heatwaves are likely to get more intense, frequent and long-lasting in the future, it becomes mandatory to develop appropriate tools for the identification of thermally hazardous environmental conditions and the assessment of potential impacts to human health (Meehl and Tebaldi 2004; Koppe et al. 2004).

A recent study demonstrated global forecasts of the Universal Thermal Climate Index (UTCI) for providing an overview of health hazards related to extreme high temperatures (Pappenberger et al. 2015). Developed by the International Society of Biometeorology (ISB) Commission 6 and the COST Action 730 through a multidisciplinary collaboration of experts, the UTCI is a bioclimate index describing the physiological heat load, called *stress*, that the human body experiences in the attempt to maintain a thermal equilibrium with the surrounding outdoor environment (Błażejczyk et al. 2013). Whereas simple heat stress indices are based exclusively on meteorological parameters such as air temperature and humidity, the UTCI is computed from an energy balance model called the UTCI-Fiala model (Fiala et al. 2012). The UTCI-Fiala model combines an advanced dynamic multi-node physiological model that includes heat and mass transfer within the body, thermoregulatory reactions of the central nervous system, and perceptual responses (Fiala et al. 2001), with a state-of-the-art temperature-adaptive clothing insulation model for outdoor climates (Havenith et al. 2012). Factors such as the human heat budget, physiology and clothing influence the ability of the human body to keep its core temperature within certain boundaries, even when the surrounding temperature is very different, and must therefore be considered when assessing the consequences of extreme high temperatures to health. Excessive exposure to heat can lead to the failure of the thermoregulatory mechanisms of the human body, which starts to produce or absorb more heat than it dissipates (Koppe et al. 2004). The core internal temperature thus starts to increase and can rise above the threshold for optimal body comfort, performance and health. High body temperatures can lead to salt and water loss through sweating (*heat exhaustion*) causing haemoconcentration, which in turn causes increases in cardiovascular diseases such as coronary and cerebral thrombosis. Very high body temperatures damage cellular structures and the thermoregulatory system, ultimately leading to death (*heat stroke*) (Keatinge 2003).

The UTCI has been evaluated and studied across different climate regions, as well as on spatial and temporal scales from the micro through to the macro (Coccolo et al. 2016). In Europe the UTCI has been applied to evaluate the bioclimatic conditions in countries such as Crete, Czech Republic, Germany, Greece, Italy, Hungary, Slovenia, Poland on a regional or city scale and across different time periods (Nemeth 2011; Novak 2013; Bleta et al. 2014a; Matzarakis et al. 2014; Błażejczyk and Błażejczyk 2014). At the same spatial domains the UTCI has been used to assess heat stress effects on mortality. In days with high UTCI values, i.e., high degree of heat stress, an increase in both death counts and number of outpatients is observed in comparison to days with no thermal stress conditions. This is found in relation to all causes of mortality and morbidity and also for specific syndromes, such as cardiovascular disease (Idzikowska 2011; Nastos and Matzarakis 2012; Bleta et al. 2014b; Urban and Kyselý 2014; Burkart et al. 2016; Błażejczyk et al. 2017). Despite these studies and the clear importance of the topic, a pan-European study where bioclimatic conditions are defined via UTCI and correlated to mortality across the whole continent has not previously been undertaken.

Within this framework the present paper aims to assess the UTCI as a heat-related health risk indicator across Europe. To achieve this, a 38-year climatology of UTCI calculated from the European Centre for Medium-Range Weather Forecasts (ECMWF) reanalysis data is presented for the summer season (June, July, August). The relationship between UTCI and mortality is then investigated at the pan-European level for the same period. Finally, results are discussed and compared with the UTCI values and number of deaths that occurred in the 2003 heatwave.

## Materials and methods

The UTCI summer climatology was computed for the 1979–2016 period at the pan-European scale using meteorological input parameters from ECMWF ERA-Interim. The relation between UTCI and mortality was assessed using death counts as provided in literature and by the Eurostat database.

### Universal Thermal Climate Index (UTCI)

For a given combination of air temperature, wind, radiation and humidity, the UTCI is defined as the air temperature of a reference environment that would elicit in the human body the same UTCI-Fiala model’s response (sweat production, shivering, skin wettedness, skin blood flow and rectal, mean skin and face temperatures) as the actual environment. The reference environment is described as a condition of calm air, i.e. wind speed 0.5 m/s at 10 m above the ground, no additional thermal irradiation, i.e. radiant temperature equal to air temperature, 50% relative humidity (capped at 20 hPa for air temperatures above 29°) where an average person walks at 4 km/h, generating a metabolic rate equal to 135 W/m^2^ ≃ 2.3 MET (Błażejczyk et al. 2013).

The offset between the air temperature of the reference environment, i.e. UTCI, and the air temperature of the real environment *T*_*a*_ depends on the actual values of *T*_*a*_, mean radiant temperature (*T*_*mrt*_), wind speed (*va*) and humidity, with the latter expressed as water vapour pressure (*vp*) or relative humidity (*RH*) (Błażejczyk et al. 2013).

For this study, the UTCI was not calculated by solving the original Fiala multi-node model as this would have been prohibitively computationally intensive and time-consuming. A faster calculation procedure, called *operational* procedure, was adopted instead (Bröde et al. 2012). The operational procedure computes the offset between the UTCI and *T*_*a*_ via a six-order polynomial equation in the environmental parameters *T*_*a*_, *T*_*mrt*_, *va*, *RH*. The procedure approximates the UTCI-Fiala model within an average root mean squared error of 1.1 °C (Bröde et al. 2012).

The UTCI is expressed in terms of an assessment scale made of ten stress levels. Each level, defined by a specific range of UTCI values, is representative of the load caused by the physiological and thermoregulatory responses of the human body when responding to the actual environmental conditions (Błażejczyk et al. 2013). As this study focuses on the summer season, seven stress levels, ranging from moderate cold stress to extreme heat stress, are considered.

### ERA-interim dataset

ECMWF ERA-Interim gridded data of 2 m temperature, 2 m dew point temperature, 10 m wind speed, thermal and solar radiation were used to compute the UTCI climatology. Solar radiation was employed to calculate the mean radiant temperature via solar height (Kántor and Unger 2011; COST Action 730 2012).

The ERA-Interim reanalysis is a global gridded dataset obtained from point-specific ground, ocean, atmosphere and satellite observations through the application of a data assimilation system based on the ECMWF Integrated Forecasting System and a 4-dimensional variational analysis (4D–Var). The ERA-Interim dataset extends from 1 January 1979 to the present date (Dee et al. 2011). It has a spatial resolution of approximately 80 km on 60 vertical levels from the surface up to 0.1 hPa and includes both 3-hourly surface parameters, describing weather, ocean-wave and land-surface conditions, and 6-hourly upper-air parameters covering the troposphere and stratosphere.

As proxy for meteorological observations, atmospheric parameters from ERA-Interim reanalysis were thus used as input to the UTCI operational code, and UTCI values calculated for each land-grid point over the 1979–2016 period. As heat-induced stress was the object of this study, the UTCI was computed with reference to the summer season, specifically from 1 June to 31 August. For each summer day the UTCI operational code was applied to ERA-Interim atmospheric parameters retrieved at five *day-time* steps, namely at 06:00, 09:00, 12:00, 15:00 and 18:00 Universal Times (UTC). The output is a set of five pan-European UTCI maps for each summer day of the 1979–2016 period. The choice to consider daytimes only for UTCI calculations is due to the definition of the UTCI, i.e. a reference condition of a person walking at 4 km/h. This condition is compatible to diurnal activities only.

### Mortality data

The relation between the UTCI and health effects associated with heat stress was assessed in terms of mortality, i.e. number of deaths. Two types of mortality datasets were considered: a monthly dataset for a summer season study over the climatological period and a daily dataset for a heatwave-specific event study.

Monthly mortality data for June, July and August were acquired from the Eurostat database for 17 European nations, namely, Austria, Belgium, Denmark, Germany, Greece, Finland, France metropolitan (France from hereafter), Iceland, Ireland, Italy, Netherlands, Norway, Portugal, Spain, Sweden, Switzerland and United Kingdom (Eurostat, © European Union, 1995–2017). The choice of using monthly data is due to the absence of a European database able to provide mortality data at finer time resolution (e.g., daily) within a common framework. The monthly mortality database provided by Eurostat spans from 1979 to 2015, which makes it consistent with the UTCI climatological period. The database refers to total mortality and does not disclose cause of death. Therefore it includes cardiovascular and respiratory deaths that may be related to heat but they are not labelled as such.

Daily mortality data for the 2003 heatwave case study were extracted for Paris from the scientific literature previously published on the topic (Vandentorren et al. 2004).

### Statistical methods

The following statistical methods were used to investigate the summer bioclimatic conditions of Europe and their relationship to mortality.

The European thermal climate was assessed at different spatial and temporal scales. At the continental scale, the time-specific UTCI maps computed from 1979 to 2016 ERA-Interim data were averaged across each month. The result is a UTCI map displaying the thermal stress at every grid point for a given day time and month. At the city scale, grid points corresponding to the location of country capitals were selected. For each point, i.e. for each capital city, the number of times a given UTCI heat stress category occurs was extrapolated and its frequency of occurrence over the summer season computed. The variability of the European thermal climate across the 38-year study period was investigated in terms of time-specific UTCI values averaged over the continent (35°N–70°N, 26°W–60°E) and over the whole summer season.

With regards to the relationship between all-cause mortality and thermal bioclimate, two approaches were adopted. For the monthly mortality dataset, nation-specific scatter diagrams were produced showing the number of all-cause deaths and the monthly average of UTCI maximum daily values. Regression lines across the whole summer season were defined by locally weighted scatterplot smoothing (LOWESS curve). For the daily mortality dataset, the degree of association between mortality and heat stress was estimated by calculating the Pearson correlation coefficient *r* between death counts and the UTCI. Specifically, Pearson correlation analysis was used to determine the relationship between the UTCI and all-cause mortality for the lag time effect, i.e. the delay between the exposure to a heat event and its associated mortality response outcome (deaths). The method used is that described in Conti et al. (2005). Briefly, for each day *i* the Pearson correlation coefficient *r* was calculated for the death counts *d*_*i*_ that occurred on that day and the average ${\widehat{UTCI}}_{n,i}$ of the time-specific UTCI values reached in *n* previous days. The exposure lag time corresponds to the day when the highest correlation value is achieved. Pearson correlation coefficients for which the probability level (*p*) is less than or equal to 0.05 are considered statistically significant.

## Results and discussion

### Thermal bioclimates in Europe

**Fig. 1:**
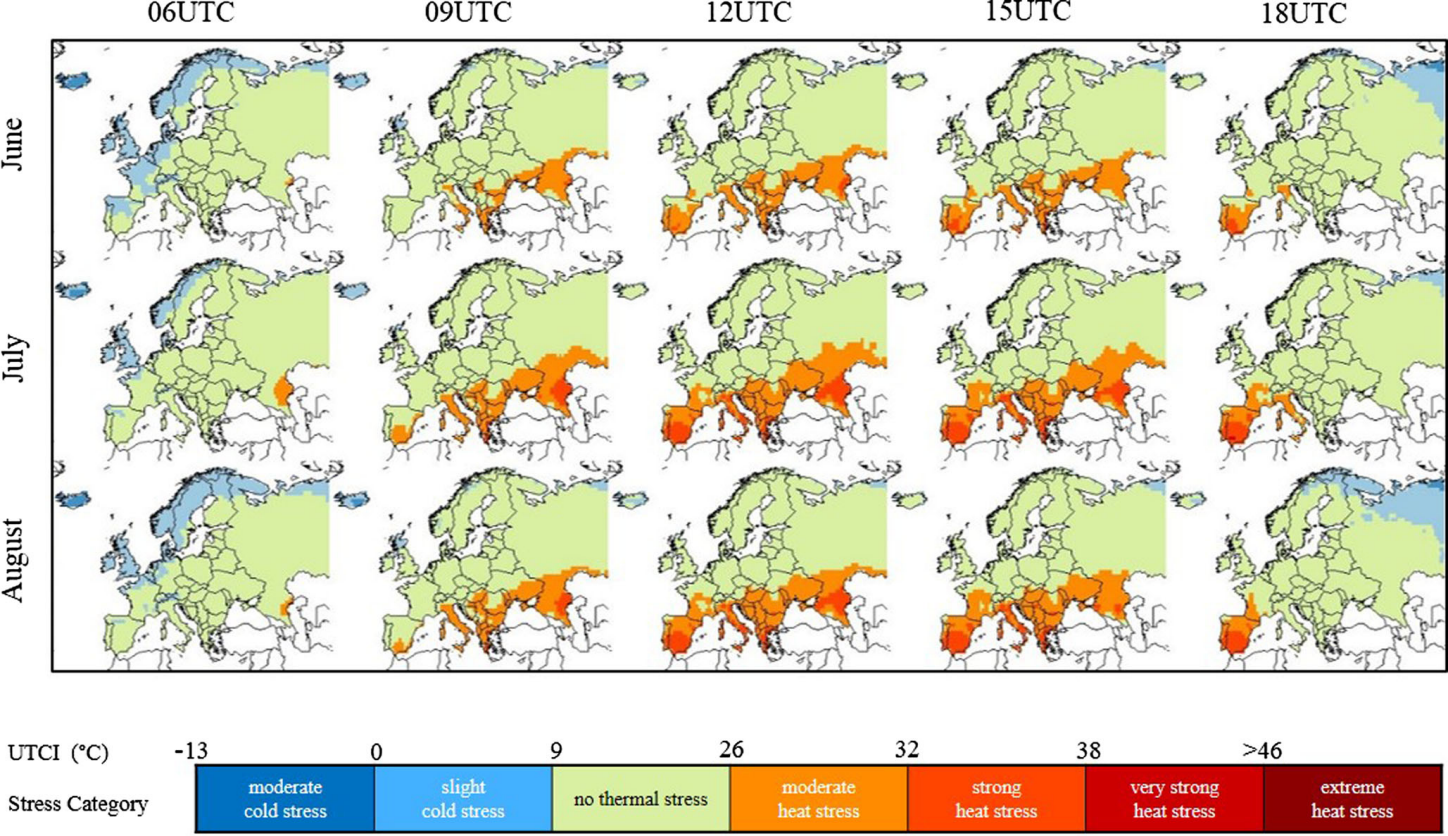
Maps of thermal bioclimates in Europe as obtained from 38 years of ERA-Interim data for the summer months and at indicated daytimes. Colour code is that of UTCI heat stress classification

Monthly-averaged time-specific UTCI maps derived from 1979 to 2016 ERA-Interim data show that the bioclimate in Europe varies in time and in space (Fig. 1). Heat stress follows a diurnal pattern with UTCI values at 06:00 or 18:00 generally lower than UTCI values at 12:00 or 15:00. Heat stress also shows a latitude gradient, with UTCI values generally increasing towards the south. As a result, two main thermal climates can be identified in Europe. One thermal climate is associated to heat stress conditions and it is predominant in the southern part of Europe, from the Iberian Peninsula to the Urals across the Mediterranean region, the Balkans and the Caucasus, where moderate and strong heat stress are achieved at central day-time hours. One thermal climate is associated to heat-neutral conditions and affects the more northern parts of Europe where on average stress due to heat load is not experienced and early/later day-time hours are characterized by cold stress instead. The definition of the two thermal climates reflects the general relationship between heat load and insolation. The UTCI has been shown to have some linear dependency on the 2 m air temperature and, through the mean radiant temperature, on the solar elevation angle and the surface solar/thermal radiation (Pappenberger et al. 2015). However, the UTCI also shows sensitivity to other atmospheric variables, namely, wind and humidity. Herein lies the added value of calculating UTCI as an indicator of heat stress.

**Fig. 2:**
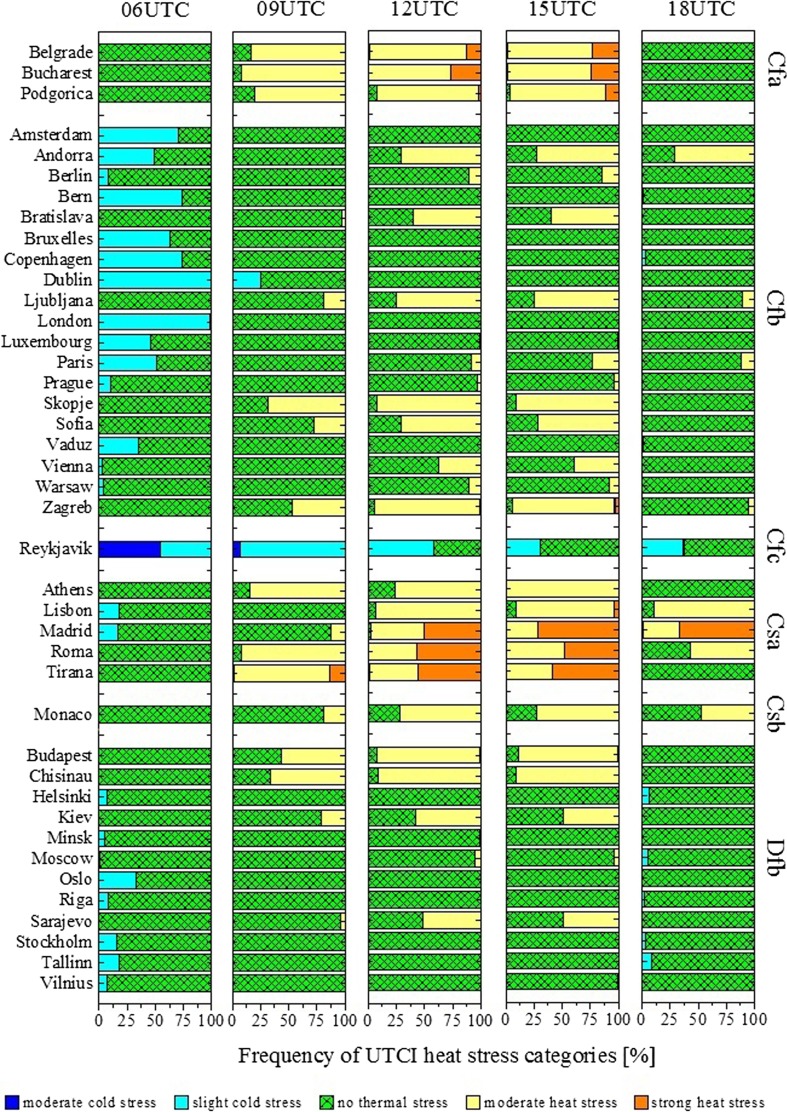
Thermal bioclimates of European capitals (1979–2016 period) at indicated daytimes. Cities are grouped according to the Köppen-Geiger climate classification

The prevalence of heat stress conditions in Southern Europe is reflected by the frequent occurrence of elevated heat stress levels in that area over the summer season. The thermal bioclimatic plots of European capitals show that the frequency of occurrence of UTCI classes differs from city to city and depends on the climate (Fig. 2). Considering central daytimes as a reference, capital cities such as Athens, Lisbon, Madrid, Roma, Tirana, Belgrade, Bucharest and Podgorica, experience either moderate or strong heat stress every day. This is in agreement with the Köppen-Geiger climate classification (Kottek et al. 2006) of the areas where those cities are located, that is, temperate climates with hot summers (*Cfa*, *Csa*). Capitals with temperate or continental climates characterized by warm summer seasons (*Cfb*, *Csb*, *Dfb*) are instead dominated by the frequent occurrence of conditions of no thermal stress or moderate heat stress. Reykjavik, characterized by a cold-summer climate (*Cfc*), is exposed to conditions of moderate cold stress up to no thermal stress.

**Fig. 3:**
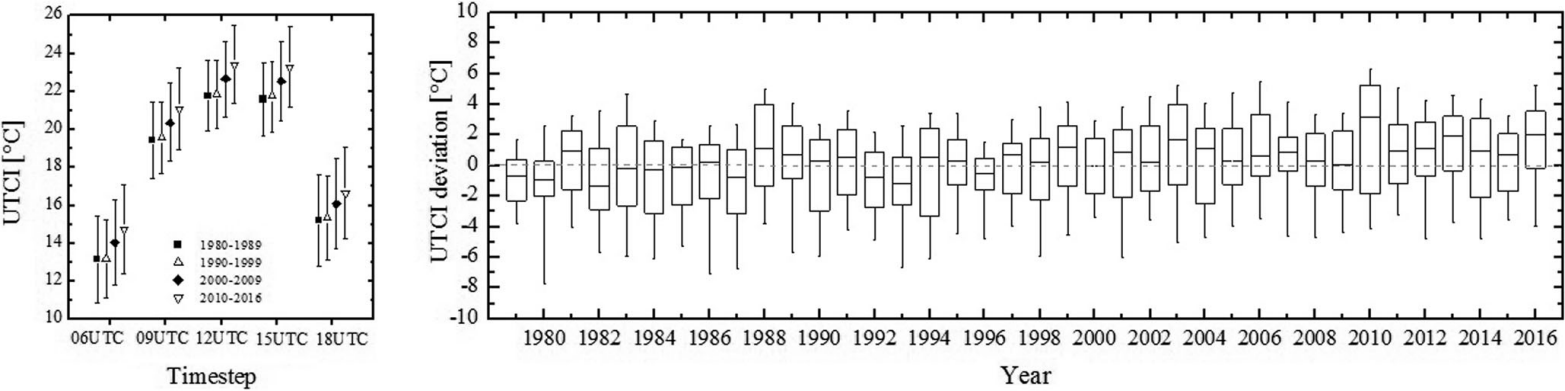
Time-dependent variability of UTCI for the summer season over the 1979–2016 period. Values are averaged over Europe. Left panel: timestep-by-timestep UTCI values per indicated periods. Error bars represent one standard deviation. Right panel: year-by-year deviation of UTCI at 12UTC from corresponding 1979–2016 summer climatological values. Solid bar is the median yearly value, boxes 25th–75th percentiles, whiskers 5th–95th percentiles

A further insight into the dependence of heat stress on daytimes is provided by a period-by-period analysis. For each of the three 10-year periods included in the study, namely 1980–1989, 1990–1999, 2000–2009 and the 7-year period 2010–2016, Europe-averaged UTCI values fall in the no thermal stress range and follow a diurnal trend which peaks at around 12:00–15:00. The intra-period variability is due to the spatial variations of UTCI across Europe (Fig. 3, left panel). As for the period-by-period variability recent summer seasons have been characterized by higher UTCI values than past ones. With respect to the UTCI reference value from the 1979–2016 climatology, the UTCI at 12UTC was about 0.5 °C colder in the 1980–1999 period, while it was 0.5 and 1 °C warmer in the 2000–2009 period and the 2010–2016 period, respectively. The differences between the periods are statistically significant. Similar deviations are observed at the other time points (data not shown). A year-by-year analysis confirms this result and attributes it to the occurrence of years, such as 2010, where UTCI median yearly values deviate positively from climatology (Fig. 3, right panel). These findings extend to the pan-European scale the results of previous studies conducted on the UTCI at the local scale, specifically the increase of UTCI values in the last three 30-year period and the occurrence of unfavourable thermal conditions in the summer months for Hungary and Central Europe (Nemeth 2011; Błażejczyk and Błażejczyk 2014).

### UTCI-mortality relation

**Fig. 4:**
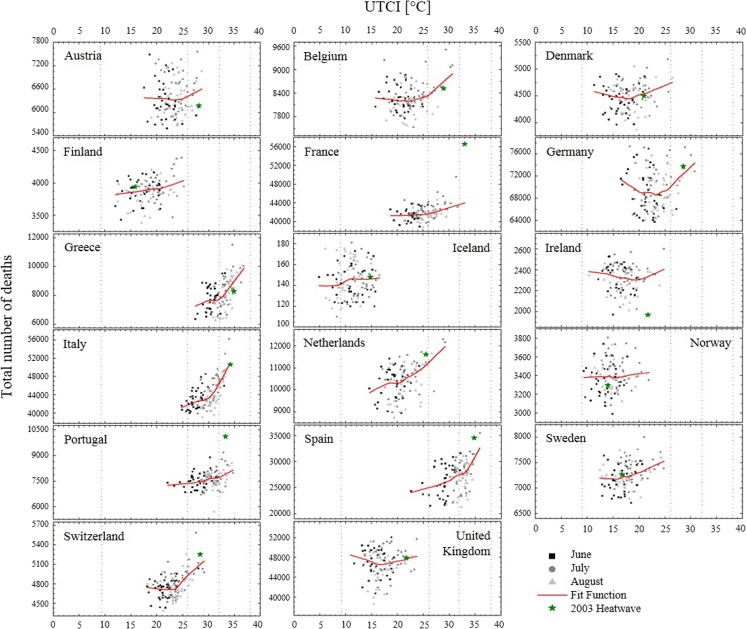
Scatter diagrams for monthly number of all-cause deaths and monthly average of UTCI daily maximum values per European country for the 1979–2015 period. Dashed lines delimitate UTCI stress categories

In order to assess the impact of heat stress on human health across Europe, the distribution of all-cause death counts at different UTCI maximum values was investigated in 17 countries (Fig. 4).

The UTCI-mortality scatterplot shows that data generally group into clusters. A *cluster* falls into one or more heat stress categories according to the country. In Denmark, Finland, Ireland, Norway, Sweden and United Kingdom, for instance, deaths are generally associated with conditions of no thermal stress. In Greece, Italy, Portugal and Spain, deaths occur mostly in conditions of moderate and strong heat stress. In Austria, Belgium, France, Germany, Netherlands and Switzerland, deaths are related to conditions of no thermal stress and moderate heat stress. For each of these groups of countries, the heat stress categories spanned by a cluster reflect the thermal bioclimate of the single country, which is the heat-neutral bioclimate for the first group, the heat-stressful bioclimate for the second group and an “intermediate” thermal bioclimate for the third group.

The influence of one heat stress category on the number of deaths is revealed by the trend of the LOWESS function used to fit the clusters. The UTCI-mortality trend depends in general on the stress category a cluster falls into. For clusters in the no thermal stress range, the association between the UTCI and mortality is either flat, i.e. the number of deaths do not increase or decrease with UTCI values (Norway, Iceland), or slightly V-shaped, i.e. the number of deaths drops as the UTCI increases up to 15–20 °C after which the number of deaths starts to rise as the UTCI increases (Denmark, Finland, Ireland, Sweden, United Kingdom). As clusters move towards UTCI domains characterized by conditions of moderate and strong heat stress, the association between UTCI and mortality assumes a more pronounced U shape or a J shape, with slopes getting steeper at about 26 and 32 °C, respectively (Austria, Belgium, France, Germany, Greece, Italy, Portugal, Spain and Switzerland). The importance of this result is twofold. First, it highlights the relevance of UTCI stress categories as ranges defined according to physiologically significant values. Second, it confirms the progressive, larger effect of heat load in the increase of mortality as conditions become more thermally stressful. This effect might be particularly notable in countries dominated by heat-stressful thermal bioclimates and strong UTCI-mortality relationships, whereas short-term extreme heat events might have greater impacts in countries characterized by more heat-neutral thermal bioclimates and weaker UTCI-mortality relationships. Further studies are needed in this regard. An analysis based on monthly mortality data may fail to capture the effect of heat-related illnesses that arise from relatively brief (day scale) but intense heatwaves. Using daily mortality data, for instance, could provide additional insights into the UTCI-mortality relationship here illustrated.

The present analysis demonstrates that the UTCI-mortality relationship is strictly connected with the thermal bioclimate to which a population is exposed and adapted. It also represents the ability of a population to cope with extreme events, such as the 2003 European heatwave.

### Case study: the heatwave of summer 2003

From June through July until mid-August, Europe experienced consecutive episodes of intense anticyclonic blockings. Areas of anomalous high pressure, firmly anchored over most of Western Europe, conveyed a very hot dry air mass up from south of the Mediterranean whilst preventing the progression of rain-bearing low pressure systems from the Atlantic Ocean. Air temperatures increased up to 12.5 °C more than seasonal average and extreme maximum temperatures were repeatedly recorded in most of the southern and central European countries (Trigo et al. 2005; García-Herrera et al. 2010).

**Fig. 5:**
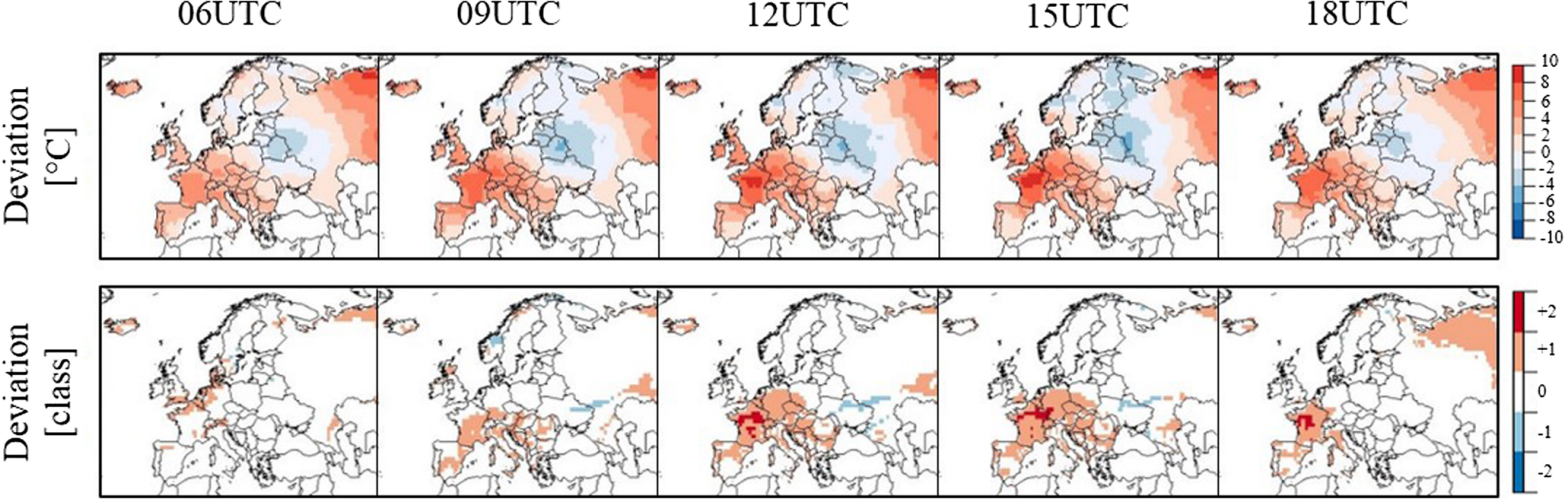
Maps of UTCI deviation from 1979-2016 climatological value expressed in degrees Celsius (upper panel) and in stress levels (lower panel) at different timesteps for August 2003

**Fig. 6:**
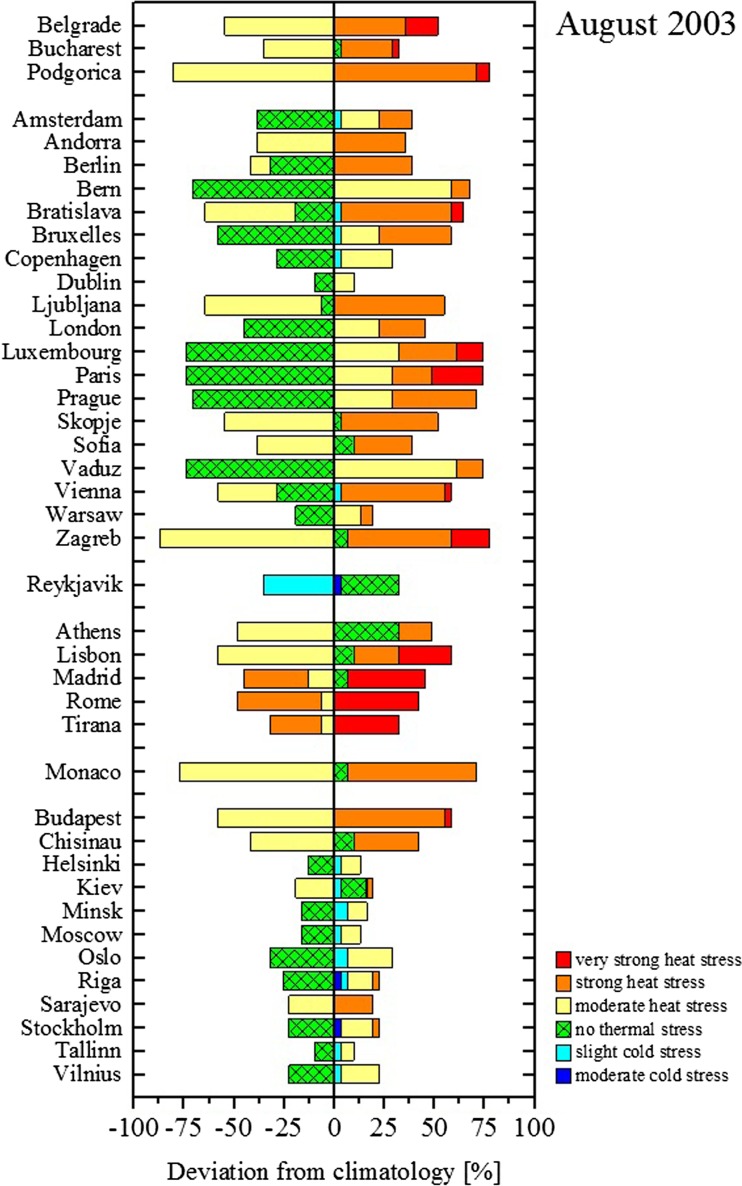
Deviation in the frequency of occurrence in heat stress classes at 12UTC per European capital for August 2003. Cities are grouped according to the Köppen-Geiger climate classification as shown in Fig. 2

The exceptionality of the 2003 heatwave is reflected in heat stress levels that affected the European bioclimatic conditions in that period. In August 2003, western and central areas experienced UTCI values up to 10 °C higher-than-seasonal average and heat stress up to 2 categories higher-than-seasonal average (Fig. 5). In France, for instance, areas usually characterized by no or moderate heat stress underwent moderate to strong heat stress for most of the daytime. In the city of Paris, the number of summer days with no thermal stress decreased by 62% as the number of days with moderate, strong and very strong stress increased by 36, 16 and 10%, respectively. The number of days with higher-than-average heat stress increased in all European capitals (Fig. 6).

**Fig. 7:**
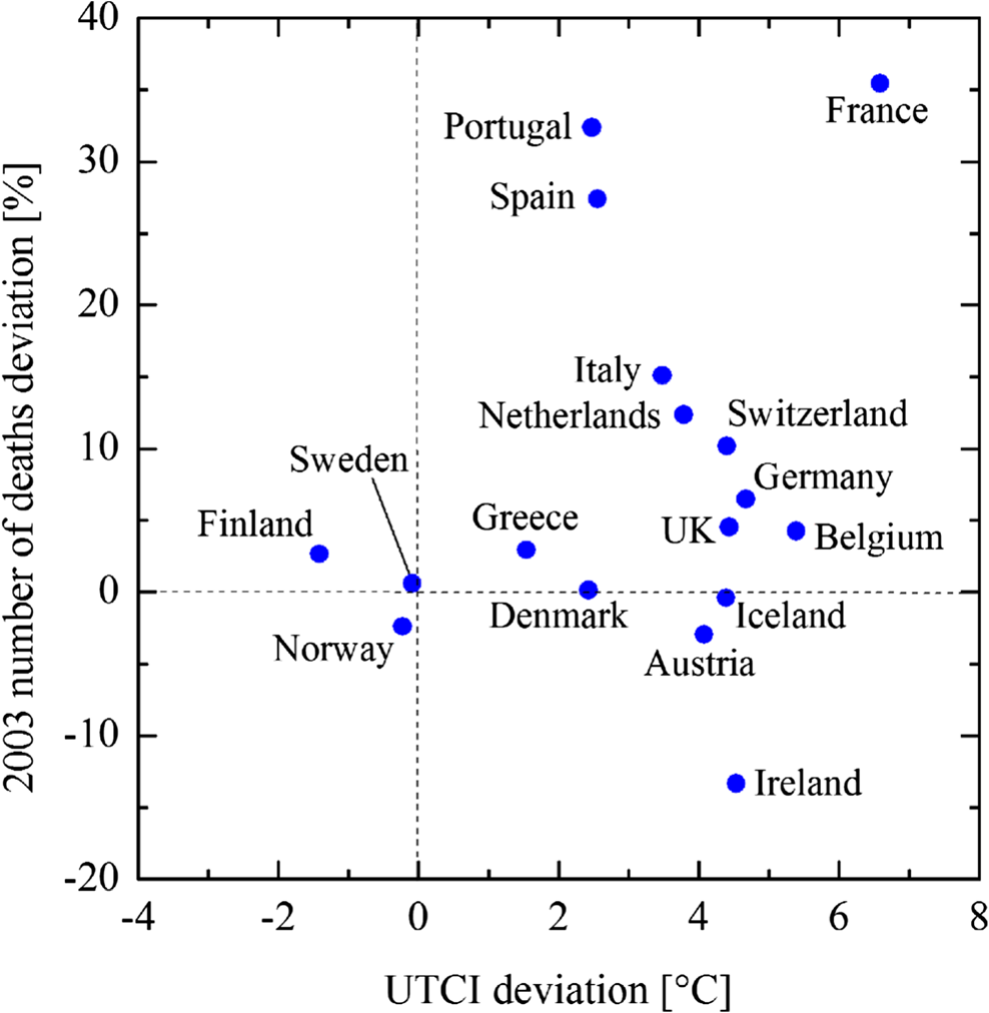
Scatterplot of UTCI deviation at 12UTC and number of deaths deviation from corresponding baselines for August 2003

The heat stress caused an unprecedented increase in mortality and morbidity, making the 2003 heatwave the deadliest natural disaster in Europe in the last 50 years (Robine et al. 2008). In August 2003 higher-than-average UTCI values were mostly associated with higher-than-average mortality, especially in France, Portugal and Spain (Fig. 7) where the heatwave exacerbated heat stress above climatological and adaptation levels. Ireland also experienced higher UTCI values, but differently from other European countries this was associated with lower death counts. This is in agreement with previous epidemiological results which state no excess deaths were reported in Ireland during the 2003 heatwave (Pascal et al. 2013).

**Fig. 8:**
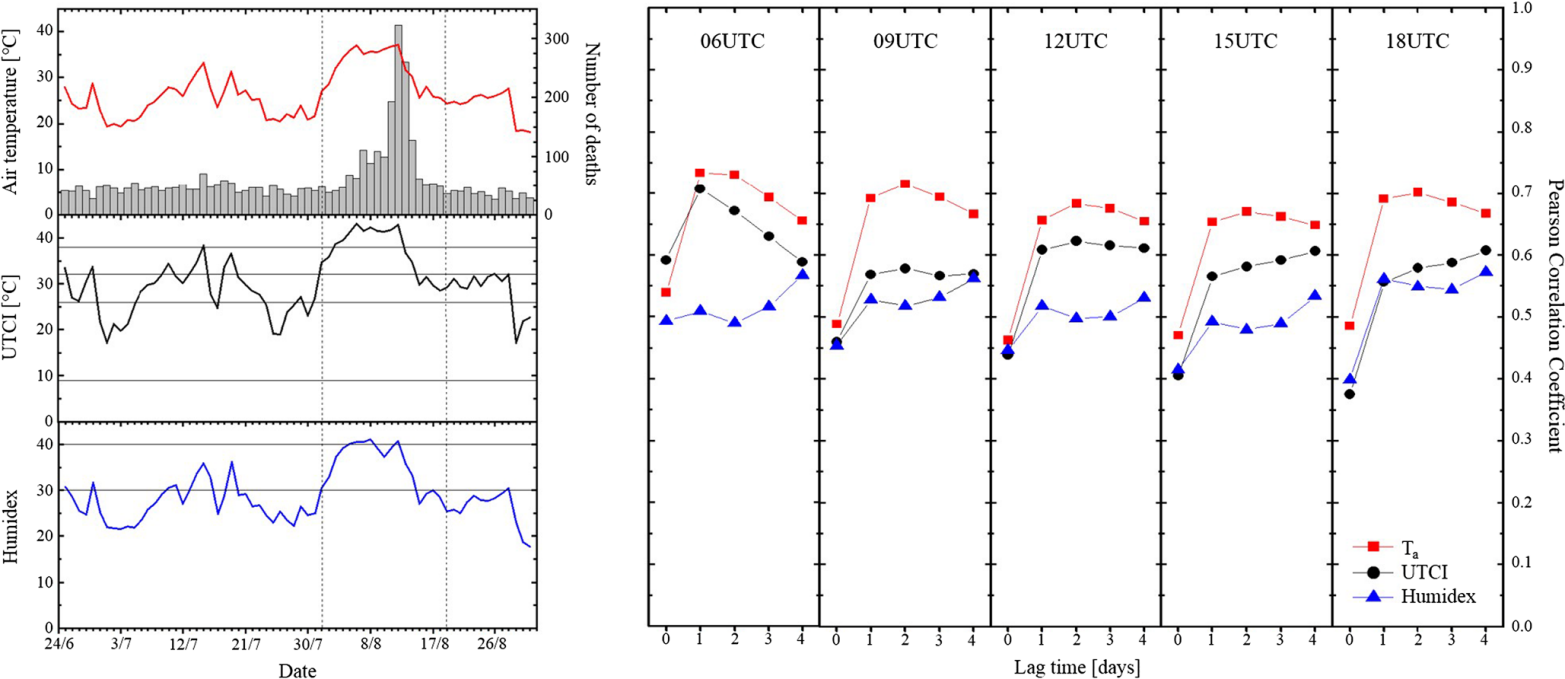
UTCI-mortality relationship in Paris during the heatwave 2003. Left panel: time series of daily death counts and daytime maximum values of 2 m air temperature, UTCI and humidex. Right panel: Pearson correlation coefficient between daily mortality and temperature, UTCI and humidex at indicated timesteps for different numbers of exposure days. The study period is 1st–19th August 2003

The impact of the 2003 heatwave on human health was particularly evident in cities such as Paris (Vandentorren et al. 2004). In the first half of August 2003, Paris experienced exceptional maximum temperatures and high minimum temperatures with the latter providing low night-time relief (Fig. 8, left panel). UTCI maximum values were above 32 °C, indicating a continuous condition of strong and very strong heat stress levels. Humidex values, computed from dewpoint temperature and relative humidity (Masterton and Richardson 1979), fell in the great discomfort category, with some peaks reaching the dangerous level. With regards to mortality, the number of daily deaths started to increase on 6th–8th August, rose to a maximum between 9th and 13th August, and returned to pre-heatwave values around 15th–16th August. The mortality peak (+ 142%, Vandentorren et al. 2004) was reached once the heatwave had become established. The fairly immediate effect of elevated temperatures on mortality is demonstrated by the fact that high correlation values (*r* > 0.65) between 2 m air temperature and daily death counts are achieved for a time lag equal to 1 to 3 days (Fig. 8, right panel). Within the same lag period the UTCI also shows a strong correlation (*r* > 0.55) with mortality, especially at 06:00 and 12:00. This result confirms that the heat stress experienced very early in the day and at central daytimes played a role in the excess number of deaths recorded in Paris during the 2003 heatwave event. It also provides insights in the correlation between UTCI and mortality with respect to 2 m temperature and humidex. At 06:00, 12:00 and 15:00 the UTCI is generally more closely associated with daily death counts than humidex. However, correlation values between UTCI and mortality are generally lower than the correlation values between 2 m air temperature and mortality. This might be due to the approximated nature of the operational procedure used to calculate UTCI or the reliability of ERA-Interim input data such as wind at finer resolution scales, i.e. at urban level. As the UTCI is by definition valid in all climates, seasons and spatiotemporal scales, and has thermo-physiologically significance in the whole range of heat exchange conditions (Błażejczyk et al. 2013), future improvements in the quality of the operational procedure as well as of UTCI’s meteorological inputs will help to shed new light in the correlation between the UTCI and mortality.

## Conclusions

In this paper the potential of the UTCI as a heat-related health risk indicator has been analysed at the pan-European level for the first time.

Using 38 years of meteorological data from the ECMWF ERA-Interim reanalysis, UTCI maps of heat stress have been computed for the summer season (June, July, August) at different day-time steps. Two thermal bioclimates have been identified across Europe: one, located at lower latitudes, characterized by the prevalence of heat stress conditions, and another, at higher latitudes, mainly dominated by a non-thermal stress state. These influence the intensity and the frequencies of occurrence of heat stress in European capitals according to their location within the European thermal bioclimates. The spatial variation of UTCI across Europe is accompanied by a time variation across decades. From 1979 onwards UTCI has increased up to 1 °C with respect to the climatological baseline, suggesting an increase in the heat stress especially in the last 15 years.

Thermal bioclimatic data have then been correlated with mortality data in 17 European countries. The nature of the relationship between the UTCI and death count is diverse (flat or V-, U-, J-shaped) and reflects the bioclimate of each country. Where heat-neutral conditions dominate the summer season mortality is either constant with respect to UTCI or reaches a minimum between 15 and 20 °C. Where heat-stressful conditions are prevalent, mortality increases as UTCI increases. The rate of increase in mortality depends on the heat stress levels and becomes evident at about 26 and 32 °C, i.e. for moderate and strong UTCI classes.

The 2003 European heatwave, with its exceptional temperatures and death toll, was used as a test bed to assess the UTCI and its relation with mortality in an extreme event. In August 2003, heat stress increased up to 2 UTCI categories above seasonal average and exposed the populations to a heat load higher than the one to which they are adapted. This caused an excess number of deaths, especially in France and its capital Paris. In Paris the mortality peak reached between 9th and 13th August was highly correlated to the very strong heat stress affecting the city 1 to 3 days before.

The findings of this analysis highlight the importance of UTCI as a bioclimatic index that not only captures the thermal bioclimatic variability of Europe but is also able to relate such variability with the effects it has on human health. Continent-wide maps of heat stress, bioclimatic diagrams of capital cities, and UTCI-mortality trends at country level provide information on which a pan-European health policy can be formulated and used by stakeholders to interpret the influence of different bioclimatic conditions to health. As awareness for disaster risk reduction is growing at the global level (Aitsi-Selmi et al. 2015), this study also reveals the potential of employing the link between UTCI and mortality in the development of an early warning systems based on the impact of heatwaves on human health.
